# Oxidised- and total non-protein bound glutathione and related thiols in gallbladder bile of patients with various gastrointestinal disorders

**DOI:** 10.1186/1471-230X-7-7

**Published:** 2007-02-23

**Authors:** Wilbert HM Peters, Annie van Schaik, Joost H Peters, Harry van Goor

**Affiliations:** 1 Department of Gastroenterolgy, Radboud University Medical Center, P.O. Box 9101, 6500 HB, Nijmegen, The Netherlands; 2 Department of General Surgery, Radboud University Medical Center, P.O. Box 9101, 6500 HB, Nijmegen, The Netherlands

## Abstract

**Background:**

Glutathione is a tripeptide composed of glutamate, cysteine and glycine, accomplishing a broad range of vital functions. Synthesis of glutathione and cysteine is performed mainly in the liver, whereas most other tissues are supplied with these thiols via sinusoidal efflux into the blood. Since canalicular efflux also occurs, thiols may be present in human bile. However, thiol composition of human gallbladder bile is largely unknown, which makes it difficult to speculate on the exact function of thiols in bile. In this study we report on the levels of non-protein bound thiols in gallbladder bile of patients with various gastrointestinal disorders.

**Methods:**

Gallbladder bile was obtained after cholecystectomy from 30 patients who were operated for pancreatic cancer, duodenal cancer, chronic pancreatitis or cholecystolithiasis. Bile was analysed for non-protein bound total- and oxidised glutathione and related thiols, by high performance liquid chromatography.

**Results:**

A more than 100-fold inter-individual variation in non-protein bound thiol levels was found in human gallbladder bile of patients with a variety of gastrointestinal disorders. Bile did contain high amounts of cysteine, whereas much lower levels of glutathione, cysteinylglycine and homocysteine were detected. Most thiols were present in their oxidised forms.

**Conclusion:**

Thiols are present in considerable amounts in human gallbladder bile of patients with various gastrointestinal disorders, levels of cysteine being much higher than those of glutathione and other thiols. Most thiols were in their oxidised forms, which may indicate the presence of considerable chemical- or oxidative stress in the patients studied here.

## Background

Glutathione is a tripeptide composed of glutamate, cysteine and glycine, that exists in a reduced (GSH) and oxidised (GSSG) form. Oxidised glutathione may also occur as protein bound glutathione (RSSG). Reduced glutathione is the active form. The most widely recognised functions of reduced glutathione are threefold: 1. maintaining the (cellular) redox status, which is essential for many vital biological functions, 2. protection of cells and tissues against toxic compounds, both of endogenous and exogenous origin and, 3. the reservoir function of cysteine, an important amino acid and building block of most proteins. Since glutathione is more stable and resistant against oxidation than cysteine, it serves as the preferred form of storage and transport of cysteine [1-3].

Glutathione is synthesized mainly in the liver and after sinusoidal efflux from liver into blood plasma, glutathione is transported, hydrolysed and taken up in tissues rich in γ-glutamyl-transpeptidase (γGT), such as kidney and lung. Thus, blood plasma serves as a transport medium for glutathione from liver to the various other tissues that are consumers of glutathione and cysteine [4].

In contrast to the hepatic sinusoidal efflux of glutathione, the role of the canalicular efflux into bile and intestine is less well understood [1]. Numerous studies on content and composition of glutathione and its metabolites in bile have been performed in bile duct canulated animals, demonstrating that the glutathione content in bile may be very high, depending on species [5]. Furthermore it was shown that glutathione in animal bile may be rapidly oxidised and hydrolysed [1]. In rats, the biliary content of glutathione was shown to be lower than, but directly proportional to the intrahepatic concentration [6]. Adjustment of toxins to rats did increase the biliary excretion of oxidised glutathione, thus indicating that biliary levels of oxidised glutathione may be a good measure of oxidative- or chemical stress [6]. This supports the main role of glutathione in detoxification. Animal studies have also suggested that glutathione is an osmotic driving force for the bile acid independent bile flow [1-3]. In addition, glutathione and its related thiols cysteine and cysteinylglycine, excreted via bile, may be taken up by the (small) intestinal epithelium, and it was suggested that this "bilio-entero-hepatic cycle" of glutathione and related thiols may function as an extra reservoir, making more "hepatic" glutathione available for sinusoidal efflux, when needed [1].

Since human bile remains in the gallbladder for several hours, composition of thiols in the human gallbladder bile, apart from inter-individual differences, may also be affected by gallbladder motility, inflammatory status, presence and number of gallbladder stones, etc. In addition, since γGT enzyme activity is high in the biliary tree of humans, composition of the gallbladder bile may differ considerably from that of primary canalicular bile [1,7].

Simultaneous measurements of the levels of total- and oxidised glutathione and related thiols in human gallbladder bile may enlarge our knowledge on the thiol status of the gallbladder, and possibly also that of the liver, of a particular patient. The thiol status of gallbladder bile may be a reflection of underlying disease, of therapeutic intervention in (biliary) disease [7] and may be a measure of the degree of oxidative- and/or chemical stress of an individual. However, hardly any data on the content of glutathione and other thiols in human gallbladder bile are available so far. Therefore we performed a pilot study on the levels and composition of non-protein bound total- and oxidised glutathione and related thiols in human gallbladder bile, obtained at surgery for pancreatic- or duodenal adenoma/carcinoma, chronic pancreatitis or cholecystolithiasis.

## Methods

### Patients

Gallbladder bile was collected after cholecystectomy from 30 patients, 19 males and 11 females, mean age 55 years [range 20–80]. Patients were operated for chronic pancreatitis (n = 7), pancreatic cancer (n = 7), duodenal cancer (n = 4), duodenal adenoma (n = 3), cholecystolithiasis (n = 7) or other reasons (n = 2) at the Department of General Surgery, Radboud University Medical Center Nijmegen, between Januari 2004 and Januari 2006. In addition to the primary diagnoses mentioned above, additional cholecystitis and/or cholecystolithiasis were found present at pathology in eight of the patients.

The study was approved by the local medical ethical review committee (CMO regio Arnhem Nijmegen). Verbal consent for the use of gallbladder specimens was obtained from each patient.

Immediately after cholecystectomy, gallbladder bile was collected by puncture of the gallbladder. Bile was transported to the laboratory and handled for assessment of total non-protein bound (both reduced and oxidised) and oxidised non-protein bound levels of cysteine (Cys and oCys), homocysteine (Hcys and oHcys), cysteinylglycine (CG and oCG) and glutathione (GSH and GSSG), essentially as described by us for the assay of thiols in whole blood [8]. In short, for assay of total thiol levels, 100 μL bile was added to 100 μL ice-cold 12% perchloric acid (PCA) containing 2.0 mM bathophenanthrolinedisulfonic acid (BPDS; Sigma Chemicals, Zwijndrecht, the Netherlands). For assay of oxidised thiols, another 100 μL bile was added to 100 μL 12% PCA containing 2.0 mM BPDS and 40 mM N-ethylmaleimide (NEM; Sigma Chemicals). After thorough mixing and centrifugation at 16,000 × g for 20 min and 4°C, supernatants were collected and stored at -80°C. Samples were analysed within one month. The excess of NEM was removed by adding 70 μL KOH (2.0 M) followed by 60 μL HCl (0.01 M in 0.3 M 3-[N-morpholino]-propanesulfonic acid buffer) to 100 μL of the sample. In the assay, 100 μL sample or standard was reduced by adding 10 μL tris(2-carboxyethyl)phospine (Fluka Chemie AG, Bornem, the Netherlands; 10% (w/v) in 0.9% sodium chloride/4.0 mM EDTA) and incubation at room temperature for 30 min. After reduction, 100 μL PCA (0.6 M with 1.0 mM EDTA) was added and samples were subsequently mixed and centrifuged for 5 min at 10,000 × g. Clear supernatant (100 μL) was incubated for 1 hour and 60°C with 240 μL reaction-mixture containing 20 μL NaOH (1.55 M), 200 μL borate buffer (125 mM K_2_B_4_O_7_·4H_2_O and 4.0 mM EDTA, pH 9.0), and 20 μL 7-fluorobenzofurazane-4-sulfonic acid (Fluka Chemie; 5 mg/mL in borate buffer). Of the derivatised sample, 20 μL was subjected to high performance liquid chromatography with fluorescent detection, using an autosampler (Model Marathon, Spark Holland), solvent delivery system (High Precision Pump model 480, Gynkotek) and fluorescent detector (Intelligent Spectrofluorometric Detector model 821-FP, Jasco) operating at excitation and emission wavelengths of 385 and 515 nm, repectively. The column used was an Inertsil ODS-2, 100 × 3 mm (5 μm particle size) and a R2 10 × 2 mm guard column, both from Chrompack (Middelburg, the Netherlands). The total-to-oxidised ratio for each thiol as a measure of oxidative stress was also calculated.

Data obtained were analysed with the Chromeleon chromatography data-system (Gynkotek, München, Germany). Thiol levels were calculated using four-point calibration curves for each thiol and were expressed in μmol/L.

All statistical analyses were performed using SPSS (version 12.0; SPSS Inc. Chicago, USA). Since multiple testing was performed, a p value of less than 0.01 was considered significant.

## Results

Median levels and ranges of the total non-protein bound and oxidised thiols, glutathione, cysteine, homocysteine and cysteinylglycine in gallbladder bile of 30 patients are given in Table 1. Median content of cysteine (Cys) was found highest (90.1 μmol/L), followed by cysteinylglycine (CG, 11.3 μmol/L) and glutathione (GSH, 7.0 μmol/L), whereas only minor amounts of homocysteine (Hcys, 0.5 μmol/L) were present in human gallbladder bile. Most thiols were present in their oxidised forms; 100%, 86% and 81% for Hcys, CG and Cys, respectively, except for glutathione which was found for only 40% in its oxidised form. This trend was also reflected in the median ratios of total non-protein bound/oxidised thiol; RCys, RHcys, RCG and RGSH, which were 1.3, 0.8, 1.2 and 1.9, respectively.

No associations were found between mean levels of any thiol and age, gender or primary diagnosis of the patients. Only tendencies for higher levels of total CG (17.2 vs. 24.0 μmol/L, p = 0.026) and oxidised CG (14.4 vs. 21.3 μmol/L, p = 0.025) were found in male vs. female patients, respectively. When the gallbladder bile thiol levels of patients without gallbladder pathology (n = 15) were compared with those of patients with cholecystitis with/or without cholecystolithiasis (n = 15), tendencies for lower total glutathione (21.4 vs. 12.7 μmol/L, p = 0.050) and lower oxidised glutathione levels (8.2 vs. 5.4 μmol/L, p = 0.067) in patients without gallbladder pathology were noticed.

## Discussion

The composition of human gallbladder bile with respect to glutathione and related thiols was studied. Earlier data on biliary excretion of glutathione or related thiols and their composition in bile were obtained almost exclusively from studies on laboratory animals, mainly rats. Except from other species differences between humans and rats, a main difference is the absence in rats of a gallbladder as a reservoir of bile. Another important difference is that most animal studies in general are performed on completely homogeneous study populations, showing relatively little variations between individual animals, which all receive the same standard diet, whereas in our study gallbladder bile of a heterogeneous patient population was investigated, which may be a main cause of variation. Glutathione was found to be the most prominent thiol present in bile of rats and mice, followed by CG and Cys, whereas in human gallbladder bile levels of Cys were highest, followed by CG and GSH, Hcys being hardly detectable. In rodents, concentrations of glutathione in bile were in the order of several mmol/L [1,5,6] whereas in human gallbladder bile, glutathione concentrations of only up to 0.1 mmol/L were found. These large differences may be either the result of species differences between humans and rodents, may be due to the various (gallbladder) disorders of the patients studied here, or may depend on the high levels of γ-glutamyl-transpeptidase in the biliary tree or gallbladder epithelium of humans, which may lead to rapid degradation of glutathione into GC and Cys.

Thiol levels of gallbladder bile showed considerable interindividual variation; several hundred-fold for Cys and CG whereas GSH values varied between 0–106 μmol/L. It should be noted once again, that the gallbladder bile investigated here was not obtained from healthy individuals but from patients with a variety of serious gastrointestinal diseases, which could contribute significantly to the huge inter-individual variation in thiol levels. Animal studies suggested that biliary concentrations of thiols may reflect the concentrations in the liver [6]. When a similar mechanism is present in humans, this would mean that hepatic concentrations are critically low in some of the patients investigated here, since very low concentrations (<2.5 μmol/L) of glutathione were measured in the gallbladder bile of 6 out of 30 individuals.

Also in contrast to the findings in animal studies are the much higher amounts of oxidised thiols, detected in human bile. Most of Cys, CG and Hcys in human bile is present in the oxidised form, whereas in animal bile most thiols are present in their reduced state. This could be explained by the fact that healthy animals were investigated, having little oxidative or chemical stress, whereas the gallbladder bile analysed here was obtained from patients after surgery for a variety of reasons. These patients were subjected to chemical stress such as anesthetics and medication, and oxidative stress as a result of the surgical intervention or the underlying disease, in most patients being cancer or (severe) inflammatory disease.

## Conclusion

This pilot study on the thiol composition of human gallbladder bile, obtained at surgery for a variety of gastrointestinal disorders, reveals that:

-Levels of cysteine are highest whereas levels of glutathione and other related thiols are much lower.

-Most thiols are in the oxidised state, possibly indicating considerable oxidative or chemical stress in the patients under study here.

-Large (several hundred-fold) inter-individual variation in thiol levels exists. Whether this large variation may be caused by the gallbladder pathology, by the health and disease status of the patient, or by biological variation between individuals, remains to be investigated.

## Competing interests

The author(s) declare that they have no competing interests.

## Authors' contributions

WHMP coordinated the research and drafted the manuscript. JHP and HvG collected the clinical samples and patient data, and revised the original draft. AvS carried out the laboratory analyses and participated in the design of the study. All authors read and approved the final manuscript.

## Pre-publication history

The pre-publication history for this paper can be accessed here:



## Figures and Tables

**Table 1:** Levels and ratios of total non-protein bound and oxidised thiols in human gallbladder bile.

Thiol	N	Median	Range
Cys (μmol/L)	30	90.1	1.4–404
oCys (μmol/L)	30	69.2	0.8–488
R Cys/oCys	30	1.3	0.6–3.8
Hcys (μmol/L)	30	0.5	0.0–2.9
oHcys (μmol/L)	30	0.6	0.0–3.2
R Hcys/oHcys*	22	0.8	0.5–5.0
CG (μmol/L)	30	11.3	0.5–84.0
oCG (μmol/L)	30	8.9	1.3–81.3
R CG/oCG	30	1.2	0.4–1.8
GSH (μmol/L)	30	7.0	0.0–106
GSSG (μmol/L)	30	2.7	0.0–34.2
R GSH/GSSG*	25	1.9	0.5–5.3

*Note that some ratios (Hcys/oHcys and GSH/GSSG) could not be calculated due to the presence of zero values.
